# Common peroneal nerve alterations associated with injuries to the posterolateral corner of the knee: how can we contribute?

**DOI:** 10.1590/0100-3984.2021.54.5e2

**Published:** 2021

**Authors:** Francisco Abaeté das Chagas-Neto

**Affiliations:** 1 Associate Researcher, Knee Imaging Research Group, Department of Orthopedic Surgery, University of Missouri Health Care, Columbia, MO, USA; Preceptor of the Musculoskeletal Imaging Sector at Hospital Antonio Prudente and at Clínica Boghos Boyadjian; Preceptor of the Radiology, Rheumatology, Orthopedics and Sports Medicine Services of the Hospital Geral de Fortaleza, Fortaleza, CE, Brazil. Email: contato@abaeteradiologia.com.br.

The common peroneal nerve is closely related to the posterolateral (ligamentous and tendinous) structures of the knee. The nerve originates from the division of the sciatic nerve in the popliteal fossa, coursing distally and laterally to emerge from the posterior face of the tendon of the biceps femoris^(1)^.

It is well known that fibular head fractures can damage the common peroneal nerve. However, traumatic involvement of the structures of the posterolateral corner of the knee and of the tendon of the biceps femoris can also injure the nerve^(1)^.

Magnetic resonance imaging (MRI) is widely used in order to assess injuries to the ligamentous and tendinous structures of the knee, including posterolateral corner injuries. The application of two-dimensional and three-dimensional techniques in MRI examinations of the knee has progressively expanded the diagnostic potential of the imaging method in the assessment of small structures in and around the joint^(2)^. In recent years, MRI became established as an important tool for the study of peripheral nerves, especially after the development of protocols including sequences that are optimized for that purpose, collectively known as magnetic resonance neurography^(3,4)^.

Chhabra et al.^(5)^ evaluated the performance of magnetic resonance neurography in evaluating the common peroneal nerve and concluded that it is a useful modality for diagnosing common peroneal neuropathy. The authors stated that the application of well-defined image classification criteria helps standardize the morphological findings on MRI and can facilitate the diagnosis of peroneal neuropathy. Those authors also proposed a classification system based on three main types of neural injury^(5)^: neuropraxia-the mildest form, involving only the myelin sheath of the nerve; axonotmesis-the intermediate form, in which there is axonal injury and Wallerian degeneration in the distal segment, without involvement of the epineurium or perineurium; and neurotmesis-the severe form, in which there is complete transection of the nerve.

In a recent study, Morris et al.^(6)^ described an interesting sign of traumatic neurotmesis of the common peroneal nerve: the lariat sign. The lariat sign consists of a proximal neural stump, completely transected, of the common peroneal nerve retracted over the preserved distal segment, just above the fibular head, making a loop in the form of a lasso.

In an original article published in this issue of Radiologia Brasileira, Marconi et al.^(7)^ discuss alterations of the common peroneal nerve, on the basis of a retrospective analysis of MRI scans of the knees in 38 patients with acute or subacute posterolateral corner injury, in comparison with scans of normal knees. Theirs was a well-designed study, in which two musculoskeletal radiologists analyzed the images, including a detailed assessment of the structures of the posterolateral corner, and classified the neural injury as neuropraxia, axonotmesis, or neurotmesis, as well as evaluating quantitative and semiquantitative parameters (signal intensity ratios: common peroneal nerve/tibial nerve and common peroneal nerve/superficial vein). The authors concluded that there is a high prevalence of common fibular nerve alterations in patients with posterolateral corner injury and that MRI has high reproducibility in the detection of such lesions. They also concluded that the use of quantitative and semiquantitative parameters can increase confidence in the diagnosis. Those conclusions are extremely important in the general routine of radiologists, because we must, from now on, systematically analyze the common peroneal nerve in detail in all MRI studies of the knee in which there are alterations in the posterolateral corner. Therefore, it is necessary for radiologists and radiology residents to be familiar with common peroneal nerve injuries and their different presentations on MRI. Armed with that knowledge, they can actively and efficiently contribute to the diagnosis and follow-up of the affected patients, thus reducing the associated morbidity and optimizing the use of imaging methods.
